# Slower is not always better: Response-time evidence clarifies the limited role of miserly information processing in the Cognitive Reflection Test

**DOI:** 10.1371/journal.pone.0186404

**Published:** 2017-11-03

**Authors:** Edward J. N. Stupple, Melanie Pitchford, Linden J. Ball, Thomas E. Hunt, Richard Steel

**Affiliations:** 1 Centre for Psychological Research, University of Derby, Derby, United Kingdom; 2 Department of Psychology, University of Bedfordshire, Luton, United Kingdom; 3 School of Psychology, University of Central Lancashire, Preston, United Kingdom; 4 School of Sport, Exercise & Health Sciences, Loughborough University, Loughborough, United Kingdom; Universita Cattolica del Sacro Cuore, ITALY

## Abstract

We report a study examining the role of ‘cognitive miserliness’ as a determinant of poor performance on the standard three-item Cognitive Reflection Test (CRT). The cognitive miserliness hypothesis proposes that people often respond incorrectly on CRT items because of an unwillingness to go beyond default, heuristic processing and invest time and effort in analytic, reflective processing. Our analysis (N = 391) focused on people’s response times to CRT items to determine whether predicted associations are evident between miserly thinking and the generation of incorrect, intuitive answers. Evidence indicated only a weak correlation between CRT response times and accuracy. Item-level analyses also failed to demonstrate predicted response-time differences between correct analytic and incorrect intuitive answers for two of the three CRT items. We question whether participants who give incorrect intuitive answers on the CRT can legitimately be termed cognitive misers and whether the three CRT items measure the same general construct.

## Introduction

Dual-process theories of thinking [1–4] propose the existence of a dissociation between intuitive, heuristic thought (which typically arises rapidly) and more effortful, analytic thought (which is typically deployed more slowly). The convention following Evans [2] is to refer to heuristic processes as ‘Type 1’ and to analytic processes as ‘Type 2’. In this paper, we use the Type 1/Type 2 and heuristic/analytic distinctions interchangeably. In testing the assumptions of dual-process theories a variety of tasks have been devised that have the potential to engender within-participant conflicts between heuristic and analytic processes. Such ‘conflict’ tasks are often associated with increased response times relative to equivalent tasks that do not engender heuristic/analytic conflicts (e.g., [5–7]). Furthermore, evidence has also clarified that the sensitivity to conflict items that is manifested in response times is often correlated with an individual’s propensity to apply analytic or calculative thinking (e.g., [8–11]).

Key examples of heuristic/analytic conflict tasks are the three items that make up the Cognitive Reflection Test (CRT [12]; see Table 1), which has taken the reasoning literature by storm over the past decade as a test-bed for examining dual-process theories. The CRT was devised to assess the ability of participants to resist tempting heuristic or ‘intuitive’ answers and to engage in analytic or ‘reflective’ reasoning to reach correct responses that conflict with intuitive responses. The most well-known item from the CRT is the ‘bat and ball’ problem, which reads as follows: “A bat and a ball cost $1.10 in total. The bat costs $1 more than the ball. How much does the ball cost? ____cents”. The common but incorrect answer to this problem is 10 cents, with this response being believed to be generated automatically by intuitive processes. In contrast, arriving at the correct response of 5 cents is generally assumed to require the inhibition of the intuitive response in favour of more careful and deliberative checking and analysis (see [13] for evidence of transcranial direct current stimulation of the dorsolateral prefrontal cortex disabling this inhibitory mechanism).

**Table 1:** The Cognitive Reflection Test.

(1) A bat and a ball cost $1.10 in total. The bat costs $1.00 more than the ball. How much does the ball cost? _____ cents. (Correct response = 5 cents; Intuitive response = 10 cents).
(2) If it takes 5 machines 5 minutes to make 5 widgets, how long would it take 100 machines to make 100 widgets? _____ minutes. (Correct response = 5 minutes; Intuitive response = 100 minutes).
(3) In a lake, there is a patch of lily pads. Every day, the patch doubles in size. If it takes 48 days for the patch to cover the entire lake, how long would it take for the patch to cover half of the lake? _____ days. (Correct response = 47 days; Intuitive response = 24 days).

The CRT has a number of key advantages for examining reasoning performance over many other tasks, which perhaps underscore its popularity. First, there are no controversies over the appropriate normative (i.e., arithmetic) standards against which to judge performance (cf. [14]), unlike the situation for most other tasks in the reasoning, judgment and decision making literature (see [15,16]). Second, the CRT is a very easy test to administer to participants and is not time-consuming for participants to complete. Third, the CRT appears to be a very consistent predictor of normative responding across many other measures of judgment and choice (e.g., see [17]).

Overall, people tend to perform poorly on the three items of the CRT, with Pennycook, Cheyne, Koehler, and Fugelsang [18] noting that both web-based and typical university samples produce means in the range of 0.5 to 1 items correct out of 3, whilst students at elite universities such as Princeton and the Massachusetts Institute of Technology yield higher means in the range of 1.5 to 2 items correct. The difficulty of the CRT is consistent with the assumptions of dual-process theories in that low scores on the test suggest that rapidly-accessible, intuitive answers derived through Type 1 processing dominate responding for a majority of reasoners, with a sufficient level of Type 2 processing only being engaged for a minority of respondents so as to enable the derivation of effective solutions by means of effortful reflection.

### Cognitive miserliness, rational thinking and the CRT

In examining the link between performance on the CRT and other cognitive abilities, researchers have frequently demonstrated positive correlations between CRT scores and normatively accurate performance on a wide range of tasks from the judgment and decision-making literature (e.g., [12,19–27]). Furthermore, Toplak et al. [27] have observed that not only does the CRT predict substantial variance in judgment and decision-making tasks that require ‘rational thinking’, but that it does so *independently* of measures of intelligence, executive functioning and thinking dispositions. This observation led Toplak et al. [27] to claim that poor scores on the CRT are an index of *cognitive miserliness* [28], that is, an unwillingness to go beyond default, heuristic processing and invest the requisite cognitive effort to solve the problem (see also [28]).

As Toplak et al. [27,28] note, the theme of cognitive miserliness in information processing has dominated judgment and decision-making research for over 40 years. As a case in point, Toplak et al. [27] refer to Kahneman and Frederick’s [29] discussion of ‘attribute substitution’ as a common mechanism used to lighten cognitive load. Such attribute substitution occurs when a person needs to assess attribute A but finds that assessing attribute B (correlated with attribute A) is cognitively easier such that they default to using attribute B instead. In other words, an easier question is substituted for a harder one. De Neys, Rossi, and Houdé [30] have explicitly evoked attribute substitution as an explanation of the bat and the ball problem on the CRT, suggesting that when participants complete tasks such as the bat and ball problem they substitute the given problem for an alternative, simpler version, in this case understanding the task as saying that “The bat costs $1” rather than what is actually stated (i.e., “The bat costs $1 *more than the ball*”).

In alignment with the cognitive miserliness hypothesis, De Neys et al. [30] demonstrated diminished confidence ratings on the bat and ball problem by those participants who gave the 10 cents response (see [31] for similar evidence using the bat and the ball problem and a ‘feeling of error’ measure). De Neys et al. [30] interpret this finding as showing that participants are not simply providing intuitive but incorrect responses to this CRT item in blissful ignorance, but rather offer the intuitive response *despite* having some awareness of its questionable nature and their possible misrepresentation of the problem. This failure to consider alternative responses or engage analytic thinking even in the face of reduced confidence in one’s answer does seem to be indicative of cognitive miserliness.

### Thinking dispositions and the CRT

Although there is reasonable evidence triangulating on the view that poor scores on the CRT index cognitive miserliness, there are also suggestions in the literature that the case is more complex than this. Indeed, despite Toplak et al.’s [27] evidence that the CRT predicts rational thinking performance independent of self-reported thinking dispositions (i.e., the *willingness* to engage Type 2 thinking), some researchers nevertheless claim that thinking dispositions may play an important causal role in CRT success. One view (e.g., [21,32–34]), is that successful CRT performance relies, at least in part, on Actively Open-Minded Thinking (e.g., [35–37]), including the search for alternative responses. For example, Campitelli and Gerrans [33] present a model of CRT performance in which both Actively Open-Minded Thinking and numeric ability are key determinants of CRT success.

Another view (e.g., [38]) is that success on the CRT indexes an ‘analytic cognitive style’, that is, a propensity to think analytically—although it is not entirely clear to what extent this idea is conceptually distinct from Toplak et al.’s [27,28] view that success on the CRT is highly predictive of ‘rational thinking’. Arguably, however, the notion of an analytic cognitive style is more generic and encompassing than that of the somewhat narrower concept of rational thinking, which adds to its appeal. Such conceptual breadth also resonates with recent evidence showing that the CRT is predictive of a wide range of outcome measures that relate to beliefs, values and skills that are not conventionally associated with research on judgment and decision making. Such outcome measures encompass religious disbelief, paranormal disbelief, less traditional moral values, enhanced scientific understanding and reasoning, belief in evolution, improved creative problem solving, less reliance on Smartphone technology as an information source and reduced receptivity to pseudo-profound bullshit (see [39] for a review).

### Numeracy skills, cognitive ability and the CRT

Toplak and colleagues’ emphasis on the value of the CRT in providing a measure of cognitive miserliness means that these researchers have, at times, seemed to downplay the extent to which the CRT might also assess so-called *mindware gaps* (e.g., the lack of the necessary cognitive rules, strategies, or belief systems to behave rationally [40]), with such mindware gaps being distinct from miserly information processing. For example, in their earlier research on the CRT, Toplak et al. [27,28] appear to view the CRT’s predictive power for rational thinking as being largely separable from constructs such as cognitive ability and intelligence as well as from underpinning mechanisms such as executive functioning and working memory. We note, for example, that whilst Toplak et al. [27] agree that mindware gaps represent an important class of reasoning error in judgment and decision-making tasks they nevertheless state that, “The potency of the CRT as a predictor of performance on heuristics-and-biases tasks *certainly does not derive from its ability to assess knowledge gaps*, because it clearly does no such thing” (p. 1284, emphasis added).

However, because the CRT consists of mathematics problems it would be curious if numeric ability was not important for successful performance. Indeed, we have already noted that the model presented by Campitelli and Gerrans [33] demonstrated that numeric ability plays a significant role in CRT performance in addition to other factors. Recent research by Sinayev and Peters [41] likewise claims that numeric ability seems to be a key mechanism that explains the observed association between CRT performance and normatively successful decision making, although they additionally note that the ability to detect and correct intuitions is also relevant to explaining the way in which the CRT is a predictor of effective decision making.

This emerging body of evidence for the ‘numeracy hypothesis’ in relation to the CRT suggests that mindware gaps may be more important for poor performance on the test than previously considered. For example, someone with poor numeracy skills would be unlikely to perform well on the CRT irrespective of their cognitive effort. The possession of appropriate mindware may not only be consequential for CRT success but also for its capacity to predict judgment and decision-making performance. These points have been supported by Stanovich, West, and Toplak [42] in their recent writing, where they directly acknowledge that CRT measures have at least moderate mindware dependence. As they note, “…even the simple bat-and-ball problem will be affected by the differential instantiation of numeracy skills. That some people find math calculations to be second nature while others do not will affect how easy the problem is” ([42] pp. 115–116).

Consistent with the view that cognitive ability and mindware are important in the CRT we also note Primi, Morsanyi, Chiesi, Donati, and Hamilton’s [43] recent proposal that the standard CRT may only be an effective measure of cognitive reflection in highly educated adults, whereas a wider range of item difficulty is needed to measure cognitive reflection in more heterogeneous samples. Thus, we suggest, the CRT may mislabel participants as cognitive misers when they struggle on the CRT when instead of being miserly they have poor levels of available working memory capacity or limited cognitive inhibition.

Thompson et al. [44] have also examined issues relating to cognitive ability and the CRT in a series of studies examining the possibility of priming deliberative thinking on CRT items by means of a ‘processing disfluency’ manipulation (see [45] [46]). In line with previous disfluency research (e.g., [45]), Thompson et al. [44] demonstrated that a degraded presentation of the CRT slowed participants down, which is suggestive of efforts at increased analytic thinking and the converse of cognitive miserliness. However, such slower responses were not generally associated with enhanced correct responding, with evidence in two experiments instead indicating that only certain sub-groups comprised of more cognitively able participants (as indexed by their SAT scores) were capable of benefitting from the disfluency manipulation. Thompson et al.’s [44] observations therefore implicate a role for cognitive ability in relation to the CRT, with higher-ability participants benefiting from the triggering of Type 2, reflective processing, although Meyer et al. [47] advance a more cautious position on this evidence.

Notwithstanding the uncertainty regarding the interaction between disfluency manipulations and SAT ability for enhanced performance on the CRT there are other recent lines of research that separately implicate cognitive ability metrics as being important for CRT success. For example, Stupple, Gale, and Richmond [48] found in two experiments that variation in working memory capacity (as measured using a composite score derived from Operation Span, Symmetry Span and Reading Span; see [49]) was a strong predictor of CRT performance, whereas the variation in response times to syllogistic reasoning problems that had been devised specifically to evoke a heuristic/analytic conflict was seen to be non-predictive of CRT success. Stupple et al.’s [48] findings run counter to a cognitive miserliness hypothesis in relation to the CRT inasmuch as increased processing times for syllogisms have previously been seen to be associated with more normative responding on these deductive arguments (e.g., [10,11]) and would therefore also be expected to be predictive of successful CRT performance.

Stupple et al. [48] argue that while cognitive miserliness may play a role in CRT performance, in order to solve the items correctly participants must nevertheless possess both the requisite working memory capacity and the relevant mindware, including numeracy skills. Stupple et al. [48] also suggest that some participants tackling the CRT might be better characterised as *cognitive wastrels* rather than cognitive misers because they appear to expend considerable cognitive effort engaged in a misdirected strategy that does not yield the correct response and that may not yield the incorrect intuitive response either.

In line with Stupple et al.’s [48] observations we also note that some researchers have recently recognised the value of scoring ‘other’ incorrect responses that are distinct from the standard ‘intuitive’ incorrect responses. For example, Pennycook et al. [18] derive various performance measures for the CRT, including the following: (1) *CRT-Reflective*, which is a participant’s total number of correct responses out of a maximum of 3; (2) *CRT-Intuitive*, which is a participant’s total number of incorrect intuitive responses out of a maximum of 3; and (3) *CRT-PI*, which is the proportion of intuitive incorrect answers out of all incorrect responses made by a participant, some of which may be non-intuitive incorrect responses (i.e., ‘other’ incorrect answers).

This latter measure has been claimed to help address statistical issues that might otherwise structurally confound CRT-Reflective and CRT-Intuitive scoring (see [18] for details). In their analysis, Pennycook et al. [18] observed that the correlation between CRT-Intuitive and a self-report measure of intuitiveness (i.e., Faith in Intuition) was not especially robust. In addition, the correlation between the CRT-PI and Faith in Intuition was not reliable as either an aggregate measure or at the item level. These results call into question the CRT as a measure of people’s tendency to rely on ‘intuitive’ responding (see [18] for further discussion, and [50], for related evidence). By implication, these findings seem to raise additional concerns about the validity of using the CRT as a measure of miserly information processing.

### Aims of the experiment

In summary, we argue that while it is likely that cognitive miserliness plays a role in CRT performance (cf. [42]), there is a growing body of evidence that the cognitive miserliness construct offers neither a sufficient account of performance on the test nor an effective explanation of the strength of the test as a predictor of what Toplak et al. ([27,28] refer to as rational thinking, or of what Pennycook et al. [18] designate as an analytic cognitive style. Furthermore, we suggest that although much work has been done to examine the predictive power of the CRT as well as modeling its underpinning cognitive factors, it is nevertheless the case that, to date, a more basic analysis has tended to be neglected. More specifically, we note that the question of whether CRT items are a good index of cognitive miserliness can be examined directly through an analysis of people’s response times to the problems and the extent to which such response times are associated with solution success.

In these latter respects we assume that a fairly pure cognitive miserliness account of the CRT that acknowledges only minimal mindware dependence (e.g., relating to numeracy skills) would predict that: (1) participants who devote the shortest times to solving CRT problems should also generally be those who minimize their task engagement and opt for intuitive but incorrect answers; and (2) participants who devote the longest times to solving the CRT problems would typically be those who avoid relying on miserly, intuitive processing and instead expend effort in analytic processing in an often successful attempt to derive correct solutions. Under these assumptions the expectation would be for a robust positive correlation between the time taken on the three CRT problems and the total number of correct solutions, with such a correlation clearly indicating convergent validity with the cognitive miserliness construct.

In contrast to these predictions, if the cognitive miserliness account of the CRT is much more limited in its explanatory scope then we would expect only a weak correlation between response times and CRT success. We note that the breaking of the link between longer response times and solution success could arise for three key reasons. First, as noted in our introductory review, it is possible that people are motivated to try to solve CRT items and not simply opt for the initial intuitive response, but that they lack the requisite mindware (e.g., numerical skills) to compute the correct answer. These individuals will have relatively long response times but might either default to an intuitive response or compute an incorrect response, either way diluting the correlation between response times and CRT success. Second, some highly numerate participants might respond quickly and accurately to most or all CRT items, this time weakening the correlation because their numeracy skills allow them to calculate quickly and accurately. Third, people might engage in time-consuming analytic processing that merely *rationalises* an incorrect, intuitive response. Because rationalisation is analytic in its intent we would not view it as being miserly information processing, yet because such rationalisation takes time whilst leading to an incorrect, intuitive response it would again reduce the correlation between response times and CRT success. We note that there is extensive evidence for rationalisation increasing response times whilst also leading to default heuristic responding for reasoning problems such as the Wason selection task (e.g., [51]; but see [52]for a more nuanced view of the evidence).

We have noted above that some participants who lack the requisite mindware to solve the CRT items might offer answers that are not consistent with either the incorrect intuitive or the correct analytic response. To investigate this issue, we aimed to conduct *item level* analyses for each CRT problem that contrasted the response times for the correct analytic and the incorrect intuitive responses to that problem with a third category of answer, that is, *incorrect non-intuitive* responses (cf. [18]). We hypothesised that these incorrect non-intuitive responses should again take longer than the incorrect intuitive responses.

## Method

### Participants

Data were collected from 391 participants, comprising 304 undergraduates studying various degree courses at Lancaster University (220 female, 84 male), and 87 undergraduates studying for a psychology degree at the University of Derby (67 female and 16 male; 4 participants declined to provide gender information). Participants were excluded if they were familiar with the task and were replaced.

### Design and materials

The three items of the Cognitive Reflection Test (Table 1) were presented to all participants via Apple Macintosh computers (Lancaster University) and PC machines (University of Derby). Responses for each item were recorded after participants had entered an answer in an on-screen field and had then clicked on a button to move to the next screen. Response times for each item reflected the total duration from its initial presentation to the submission of a response.

### Procedure

Ethical clearance was granted through the local ethics committees at Lancaster University and Derby University prior to commencement of data collection. We confirm that participants provided written consent and this consent procedure was approved by the local ethics committees at both Lancaster University and Derby University. Items were presented by computer one at a time in a randomized order. On completion of all of the items participants were thanked for taking part in the experiment and were given a printed debrief sheet. Any questions arising from the experiment or the debrief sheet were answered prior to participants departing the experiment.

### Data analysis

Data were analysed using SPSS Version 24. Response time data were positively skewed and were therefore log10 transformed so as to meet the necessary requirements for parametric data analysis using regression. This regression analysis used the log10 transformed response times for each of the three separate CRT items to predict the overall CRT performance score. We further note that for the item-based comparisons of response times for the different response types the data failed to meet the assumptions for parametric analysis in terms of the absence of either normal distributions or equal group sizes. As a consequence, we analysed these data using non-parametric Kruskal Wallace tests and applied Bonferroni-adjusted Mann-Whitney follow-up tests.

## Results

**Fig 1:**
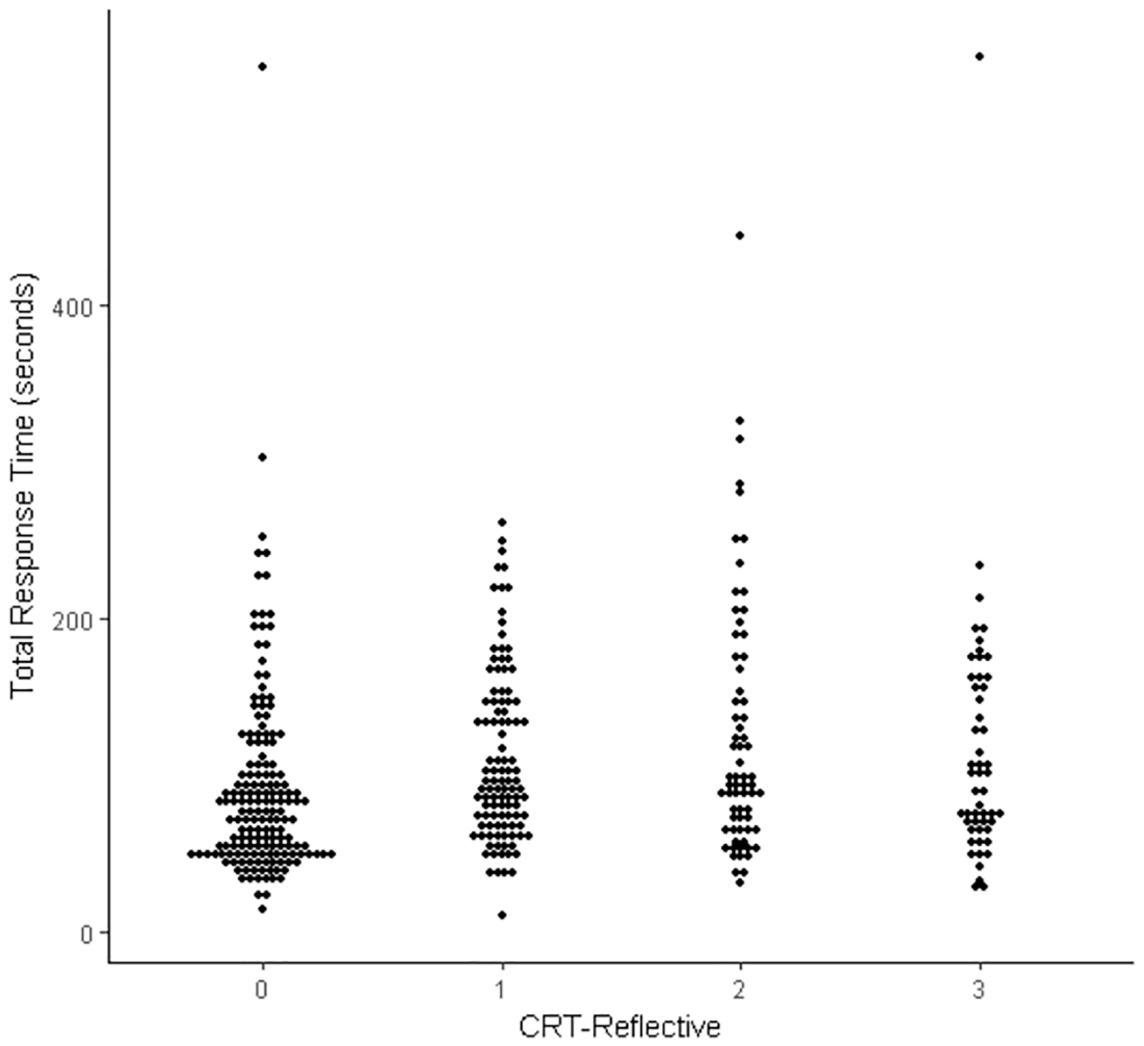
Dot plot of the relationship between CRT-Reflective scores and total response times (natural data in seconds).

The first analysis examined the association between the sum of participants’ response times (log_10_ transformed to correct for violations of normality) for the three CRT items and the total number of items responded to correctly (i.e., the CRT-Reflective measure described by Pennycook et al [18]). The correlation was positive and significant, *r* = .181, *p* < .001 (N = 391), as predicted by the cognitive miserliness account, but the relationship was relatively small, indicating that longer response times were only weakly associated with better CRT performance. Note that the correlation between participants’ *mean* response times for the three CRT items and the total number of items responded to correctly was equivalent to the one reported, which involved *total* response times for the three CRT items. This relatively weak relationship between correct performance and response times is readily apparent when CRT-Reflective scores and total response-time data are depicted graphically as a dot plot (see Fig 1).

As a follow-up analysis a multiple regression was conducted to test the contribution that log_10_ transformed response times for each of the CRT items made to predicting overall correct responding for the three problems (i.e., the CRT-Reflective score). The regression model was highly significant (see Table 2), but standardised regression coefficients for each problem type showed differing relationships with overall correct responding. For the bat and ball item, longer response times were associated with increased overall performance, indicating that increased response times to this item predicted correct responding overall. However, response times to the widget problem did not significantly predict correct responses. Finally, the response times for the lily pads problem indicated that *reduced* response times were significantly associated with more correct responses overall.

**Table 2:** Multiple regression of log_10_ transformed response times for CRT items as predictors of the CRT-Reflective score.

*Regression Model*	*R* = .344, *R*^*2*^_*adj*_ = .111	*F*(3, 387) = 17.28, *p* < .001	
*Predictors*	*Standardized Beta*	*Unstandardized Beta*	
Bat and Ball RT	0.344	1.143	*t* = 6.35, p < .001
Widget RT	0.073	0.230	*t* = 1.34, p = .182
Lily Pad RT	-0.155	-0.572	*t* = -2.86, p = .004

**Fig 2:**
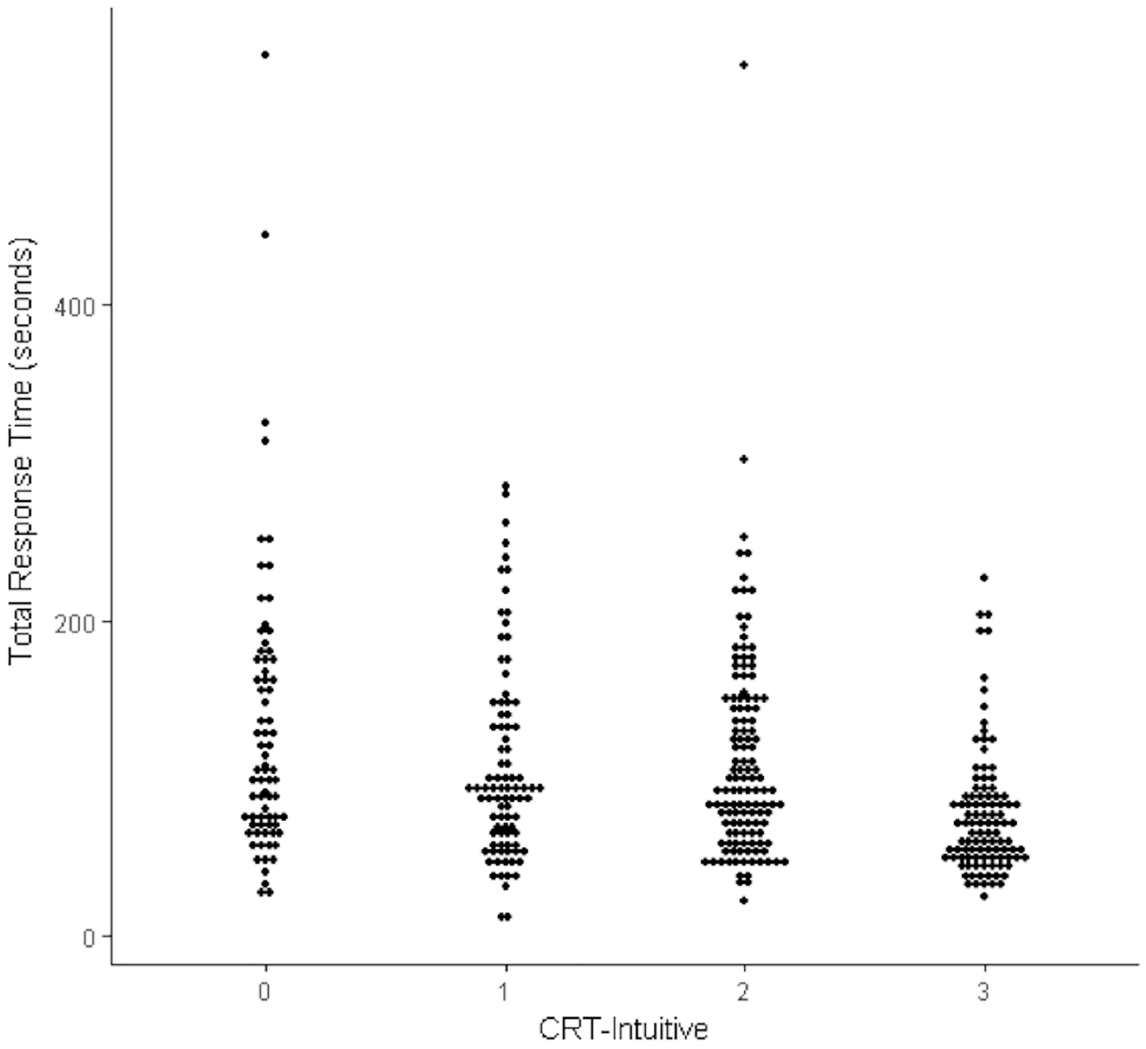
Dot plot of the relationship between CRT-Intuitive scores and total response times (natural data in seconds).

Pennycook et al. [18] propose that to examine miserly thinking in the CRT it is best to calculate and examine intuitiveness scores (see Fig 2 for a dot plot depicting the relationship between CRT-Intuitive scores and total response times). A further regression was conducted that was structurally equivalent to that reported above, but which used Pennycook et al.’s CRT-Intuitive score as the dependent variable, which is a participant’s total number of incorrect intuitive responses out of a maximum of 3. This model was again highly significant (see Table 3). For the bat and ball item, shorter response times were associated with increased intuitive responding overall. However, response times to the widget problem, and response times for the lily pads problem, did not significantly predict intuitive responding.

**Table 3:** Multiple regression of log_10_ transformed response times for CRT items as predictors of the CRT-Intuitive score.

*Regression Model*	*R* = .337, R^*2*^_*adj*_ = .107	*F* (3, 387) = 16.52, *p* < .001	
*Predictors*	*Standardized Beta*	*Unstandardized Beta*	
Bat and Ball RT	-0.330	-1.140	*t* = -6.08, *p <* .001
Widget RT	-0.064	-0.230	*t* = -1.17, *p* = .241
Lily Pad RT	0.074	0.274	*t* = 1.37, *p* = .173

The readily apparent asymmetry between the regression models for the CRT-Reflective score and the CRT-Intuitive score suggest that *incorrect but non-intuitive* responses may be very important in explaining the relationship between response times and performance on the CRT. To examine this issue further, we examined the response times for each of the three CRT problems separately, having first designated a participant’s response to the item as being one of three possible types: (1) correct analytic; (2) incorrect intuitive; or (3) incorrect non-intuitive. Due to uneven sample sizes across groups non-parametric ANOVAs were conducted. Median response times for each type of answer for the CRT items are shown in Table 4, whilst Table 5 shows the frequency of each type of response across the CRT items.

**Table 4:** Median response times in seconds (interquartile range in parenthesis) as a function of response type and CRT problem.

CRT Problem	Response Type		
	Correct Analytic	Incorrect Intuitive	Incorrect Non-Intuitive
Bat and Ball	34.97 (36.64)	19.38 (14.96)	27.31 (42.74)
Widget	28.81 (32.38)	24.29 (20.98)	34.30 (44.20)
Lily Pads	28.19 (26.16)	24.95 (22.92)	50.63 (53.41)

**Table 5:** Frequency of response type as a function of CRT problem (N = 391).

CRT Problem	Response Type		
	Correct Analytic	Incorrect Intuitive	Incorrect Non-Intuitive
Bat and Ball	117	259	15
Widget	119	209	63
Lily Pads	163	179	49

**Fig 3:**
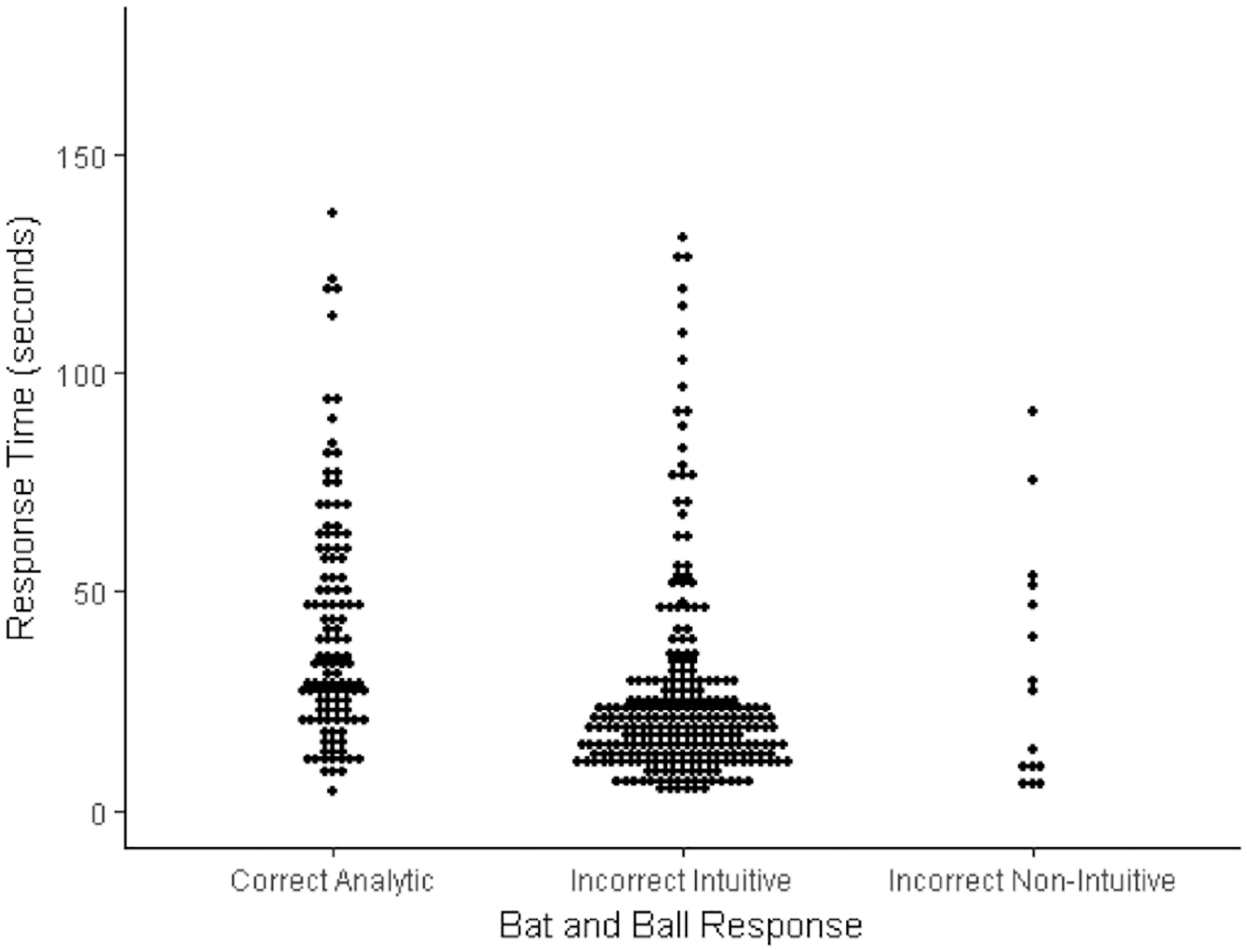
Dot plot of the relationship between response categories and response times (natural data in seconds) for the bat and ball problem.

For the bat and ball problem incorrect intuitive answers were the most common (259 responses) and incorrect non-intuitive answers were the least common (just 15 responses). In terms of the response-time analysis, this demonstrated a highly significant difference across response types, Kruskal Wallace *H* = 49.99, *df* = 2, *p* < .001. Post hoc Mann Whitney tests (Bonferroni adjusted alpha = .017) indicated that the difference in response-times for correct analytic and incorrect intuitive responses was highly significant and in line with cognitive miser predictions (*p* < .001). Mann Whitney tests showed that there was no significant difference either between correct analytic and incorrect non-intuitive responses (*p* = .09) or between incorrect intuitive and incorrect non-intuitive responses (*p* = .86), although these analyses were potentially compromised by the small number of responses in the incorrect non-intuitive category. In sum, the data for the bat and ball problem indicate that correct analytic responses took longer to derive than incorrect intuitive responses (cf. Fig 3), which aligns with predictions relating to cognitive miserliness dominating responding on this CRT item given that the incorrect intuitive answers were also more frequent.

**Fig 4:**
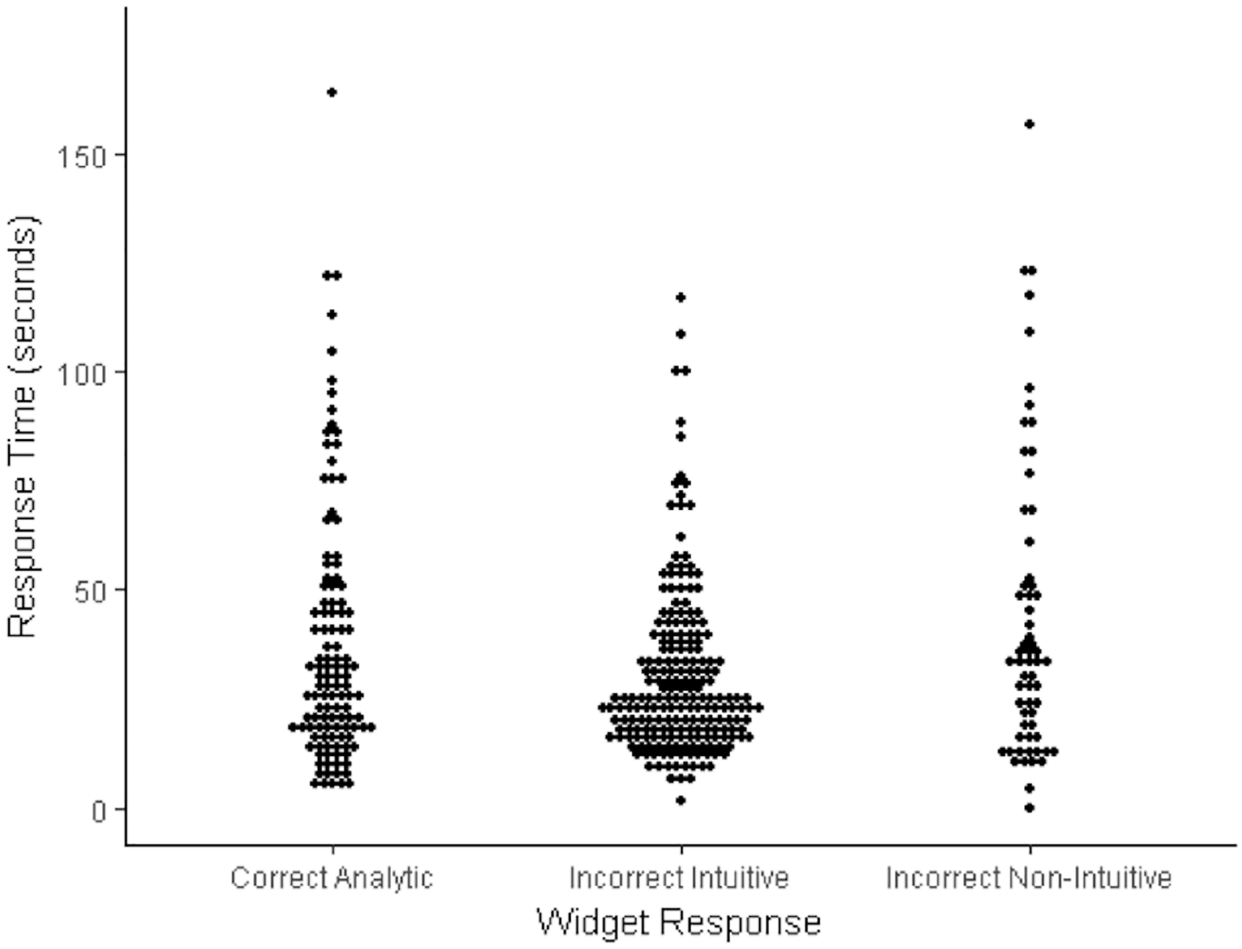
Dot plot of the relationship between response categories and response times (natural data in seconds) for the widget problem.

For the widget problem incorrect intuitive answers were again the most common (209 responses) and incorrect non-intuitive answers were the least common (63 responses), although proportionally the latter were more prevalent for the widget problem than for other CRT items, suggesting that with this problem a fairly sizeable subset of participants were attempting to derive an analytic solution rather than defaulting to an intuitive one, but were nevertheless unable to derive a correct response. The response-time analysis for the widget problem demonstrated a significant difference across response types, Kruskal Wallace *H* = 7.25, *df* = 2, *p* = .027 (see also Fig 4). However, post hoc Mann Whitney tests (Bonferroni adjusted threshold alpha = .017) showed that this difference was not significant between correct analytic and incorrect intuitive responses (*p* = .09), which runs counter to cognitive miserliness predictions. There was also no difference in response times between correct analytic and incorrect non-intuitive responses (*p* = .29), although incorrect non-intuitive responses did take reliably longer than incorrect intuitive responses (*p* = .013).

**Fig 5:**
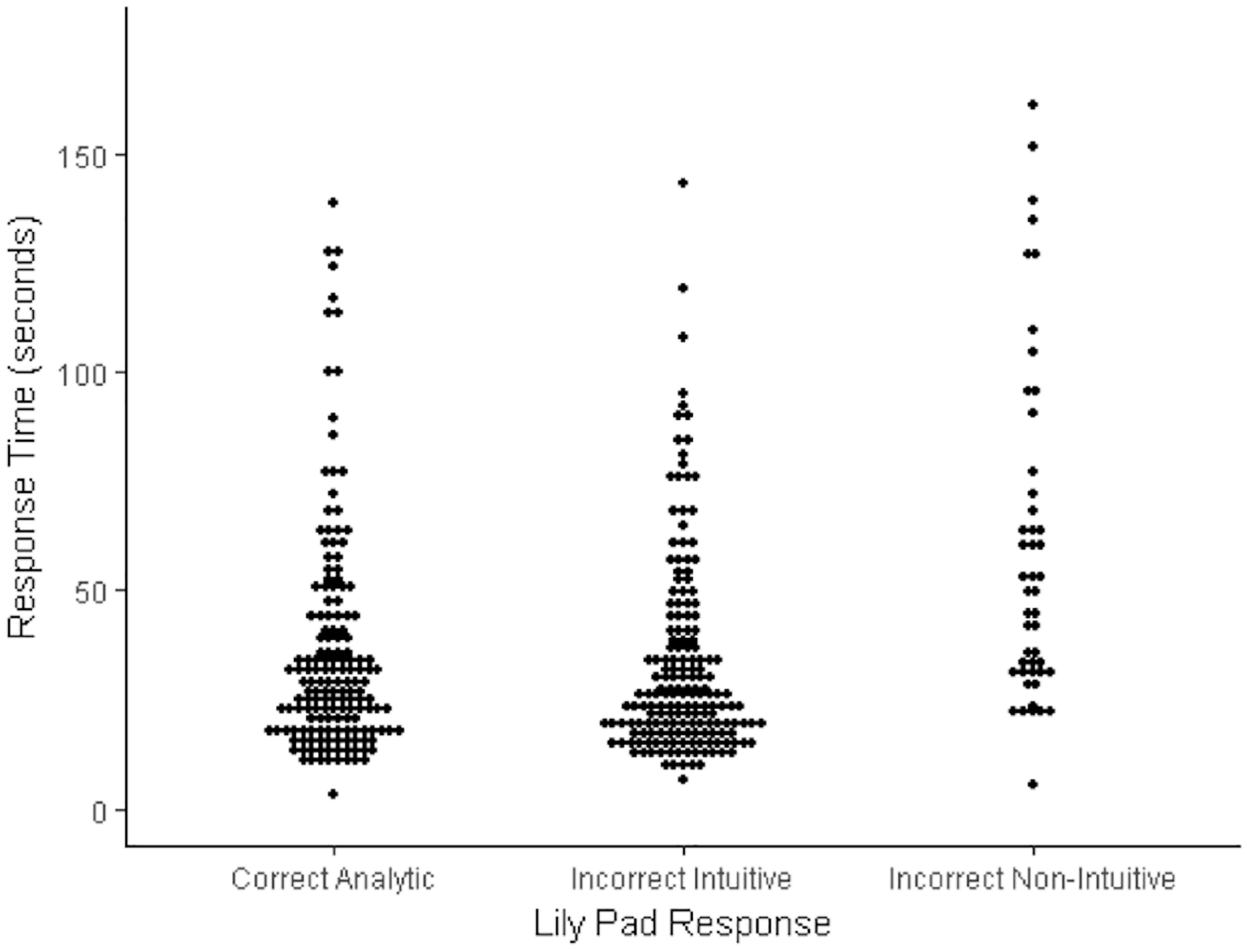
Dot plot of the relationship between response categories and response times (natural data in seconds) for the lily pads problem.

For the lily pads problem, incorrect intuitive answers were again the most common (179 responses), although correct analytic responses were fairly frequent (163 responses) and combined with incorrect non-intuitive responses (49 responses) indicated that overall a majority of participants were attempting to derive analytic solutions for this item. Other researchers have likewise observed that the lily pads problem tends to be evoke more analytic responding that the other two items and is somewhat easier (e.g., [18,33]). The response-time analysis for the lily pads problem again revealed a significant difference across response types, Kruskal Wallace *H* = 31.61, *df* = 2, *p* < .001 (see also Fig 5). Post hoc Mann Whitney tests (Bonferroni adjusted threshold alpha = .017) demonstrated that this difference was not significant between correct analytic and incorrect intuitive responses (*p* = .25). However, incorrect non-intuitive responses took reliably longer than both incorrect intuitive responses (*p* < .001) and correct analytic responses (*p* < .001).

Finally, we examined the internal reliability of the CRT. The items showed an 'unacceptable' level of reliability, Cronbach’s ά = .47.

## Discussion

The results of this study reveal a weak but reliable correlation between CRT response times and overall CRT accuracy. Although this evidence does not support a pure cognitive miserliness account of CRT performance, it does, nevertheless, suggest that miserly information processing is part of an explanation of solution errors on the CRT. If cognitive miserliness was the primary explanation of CRT performance then one would expect a stronger pattern of positive association between CRT response times and accuracy rates, with correct responses taking time to be produced via analytic, Type 2 processing, and with incorrect intuitive responses arising quickly via heuristic, Type 1 processing. Instead, our data suggest that several factors are at play when participants complete the CRT.

Further evidence against a pure cognitive miserliness hypothesis emerged from examining individual CRT items. Regression analyses showed that although response times for the bat and ball problem predicted overall successful CRT performance and were therefore consistent with a cognitive miser account, this was not so for the widget problem. Most notably, *faster* responses to the lily pads problem were predictive of *better* overall CRT performance, in opposition to what might be expected from a cognitive miser account. Furthermore, the item-level analyses contrasting response times across response types (incorrect intuitive, incorrect non-intuitive and correct analytic) also failed to demonstrate predicted differences between correct analytic responses and incorrect intuitive responses for two of the three CRT problems, with the exception again being the bat and ball problem.

Because these latter comparisons are not contaminated by responses times arising when people respond non-intuitively but incorrectly, they represent a more clear-cut test of the cognitive miserliness hypothesis than the weak correlation between response times and accuracy rates. The observation that the predicted response-time difference between correct analytic responses and incorrect intuitive responses was only evident for the bat and ball problem raises questions about the validity of a pure cognitive miserliness explanation of poor CRT performance. We note that a recent study by Travers, Rolinson, and Feeney [53], which examined response times using an adapted version of Primi et al.’s [43] eight-item CRT, found that participants took significantly longer to evaluate correct analytic answers relative to incorrect intuitive ones when response-time data were analyzed at an aggregate level. However, since an item-level analysis was not presented for this comparison it is uncertain to what extent the observed effect was driven by differences arising for perhaps just a few items, such as the bat and ball problem, as in the present study.

Travers et al.’s [53] study presents a fascinating and detailed analysis of responding on conflict and non-conflict versions of the CRT using a mouse-tracking methodology to capture the time-course of processing. We note, however, that there are some non-standard aspects of their mouse-tracking paradigm, which may render it difficult to draw clear-cut comparisons with the standard presentation of the CRT, including the presentation of four response options per problem. There are also more general concerns with what mouse-tracking data can legitimately reveal regarding reasoning processes, which arise because of inherent methodological artifacts that seriously bias response-time effects in ways that ultimately confound theoretical interpretation, even producing effects that are opposite to these found using more sensitive attentional measures such as eye-tracking analysis (for relevant discussion see [51,54–56].

Our data not only question whether participants who give incorrect intuitive answers to the CRT can simply be referred to as cognitive misers, but also indicate that a sizeable minority of incorrect responders (especially on the widget and lily pads problems) did not produce predicted intuitive answers. This observation again suggests that such individuals cannot be labeled as cognitive misers since they are presumably engaging in analytic thinking, albeit analytic thinking that is not delivering a correct solution. Interestingly, participants who generated the incorrect non-intuitive responses for the widget and lily pads problems also demonstrated the longest response times for these items and may be best categorized as *cognitive wastrels* [48], since they seem to be trying to compute a response whilst not possessing sufficient cognitive abilities—numeric or otherwise—to derive an effective solution.

In this latter respect the present evidence suggests that some aspects of poor CRT performance derive from a failure to compute the normative response because of mindware gaps (e.g., in basic mathematical skills), which means that it is possible that the CRT also has the capacity to assess such gaps on various other judgment and decision making tasks employed in heuristics-and-biases research (e.g., tasks also involving fundamental numerical skills such as ratio-bias problems; e.g., [57]). Indeed, we again note Sinayev and Peters’ [41] evidence that numeric ability is one of the key factors that explains the standard association observed between CRT performance and successful responses on judgment and decision-making tasks.

Returning to our finding that two CRT items failed to engender predicted response-time differences between correct analytic responses and incorrect intuitive responses—as expected by a cognitive miserliness account—we suggest that at the very least these data raise concerns about the internal reliability of the CRT in terms of whether the items measure the same general construct. The Cronbach's alpha we observed was at an unacceptable level and not dissimilar to that seen in previous studies (e.g., Teovanović and colleagues [58], reported a Cronbach’s ά of .39). Indeed, Toplak et al. [28] have themselves commented that from the standpoint of reliability “three items is obviously too few” (p. 150), which has, in part, inspired them to validate a seven-item version of the CRT. We also note Teovanović et al.’s [58] research examining the factor structure of a range of cognitive biases, which found that while the standard three-item CRT correlated well with some of the individual tasks it did not load significantly onto any factors identified as explaining variance in these cognitive biases. Teovanović et al. argue that this outcome might be because of a lack of internal consistency between CRT items.

If different CRT items are measuring different cognitive and dispositional constructs, then this has major implications for how the CRT is interpreted and utilised and warrants further investigation. In this respect, we applaud the development of extended versions of the CRT ([28,43,59]) given that these have a broader focus, increase the range of possible scores, reduce the confounding influence of numeracy and include items that some less cognitively able individuals can solve. Our own data, which indicate that some participants may have engaged in misdirected analytic thinking on standard CRT items, suggest that the efforts by Primi et al. [43] to measure reflective thinking in less able participants are especially welcome.

In terms of the internal reliability of the standard three-item CRT, we suggest that the bat and ball problem appears to be the most convincing candidate for measuring cognitive miserliness, with the other two items appearing not to measure cognitive miserliness at all. Certainly, for the lily pads problem there is little or no calculation that needs to be done to produce the correct solution. The lily pads doubling in area each day simply requires the realization that the lily pads would be at half their final extent the day before the lake is completely covered, which contrasts markedly with the bat and ball problem, where realizing that 10 cents is not the correct answer requires the application of some (albeit basic) algebra, whereby candidate values for the ball are tested. We would argue that this is an example of the kind of sustained ‘decoupling’ that Stanovich and Toplak [60] describe as a necessary criterion for Type 2 processing. It is also noteworthy in relation to the lily pads problem that if participants do engage in a misdirected calculative strategy from the outset then they often invest a substantial amount of time in reaching an incorrect answer. In this respect, the lily pads problem may best be categorized as an ‘insight’ problem that hinges on a restructured representation of the given information. Indeed, an earlier version of the problem studied by Schooler, Ohlsson, and Brooks [61] was described in such terms.

A final question to address given our concerns with the reliability of CRT items as a measure of cognitive miserliness relates to the matter of what participants are doing when they take a median of around 19 to 25 seconds on these items only thence to generate an incorrect intuitive response. We note that this important question is not directly addressed by Travers et al. [53] in relation to their findings regarding the time-course of processing on the CRT. This is despite their observation that the average time to provide the incorrect intuitive response to CRT items involving a heuristic/analytic conflict was a lengthy 21 seconds, which is closely aligned with the median response time observed in the present study for incorrect intuitive answers. These relatively slow responses seem to support aspects of De Neys et al.’s (e.g., [30]) theoretical perspective, which proposes that although participants may be unconvinced by their initial, intuitive answer to a CRT item such that analytic Type 2 reasoning might be triggered, they may also lack the requisite mindware to solve the problem and still default to an intuitive response without engaging Type 2 analytic thinking, albeit with a sense of doubt as to the validity of their answer. Presumably, this uncertainty takes time to be resolved, thereby extending response times even though the eventual answer that is proffered in the incorrect intuitive one. It is also possible that these participants *do* strive to resolve the uncertainly through analytic processing (which takes time), but because of the inherent processing demands of this analytic thinking the intuitive response is defaulted to as a form of ‘computational escape hatch’ [62–64]. In explaining the long response times for incorrect intuitive responses, we also reiterate here the possibility that people might engage in time-consuming analytic processing that ends up merely *rationalising* the intuitive response.

We note that all of these explanations for relatively long processing times for incorrect intuitive answers on the CRT arise through analytic, Type 2 processes and *not* because of miserly information processing. More generally, it is important to note that the relationship between responses and response times is not always straightforward and that ‘slowness’ has been argued to be an incidental correlate of Type 2 processing rather than a necessary criterion [3,60]. However, it is difficult to argue that when a participant is investing more time in responding to an item they are being miserly, even if they are attempting to come up with a post hoc rationale for an erroneous response. Similarly, it is difficult to see how responses derived from autonomous processes might be consistently slower than those derived from the sustained decoupling of problems from hypothetical solutions. It should also be noted that the dual-process explanation of the CRT, while dominant in the literature, is not universally endorsed (see [65,66], for critical perspectives on dual-process theory).

In conclusion, we contend that although the CRT is widely assumed to be an effective measure of cognitive miserliness, our findings indicate that it is prudent not to make this assumption uncritically. Indeed, we see grounds for viewing two of the three items used on the CRT as being poor measures of cognitive miserliness, which may undermine the internal reliability of the CRT as a whole. We would suggest that researchers using the CRT to measure miserliness would do well to find some approach to eliminate spurious variance arising from responses that are neither correct analytic answers nor incorrect intuitive answers, since in many cases these incorrect non-intuitive responses are not miserly. In this respect, we welcome the emergence of alternative scoring approaches in the CRT literature [18,41]. Moreover, we consider it to be judicious for researchers to continue to use measures of cognitive disposition such as the Need for Cognition Scale [67], the Rational Experiential Inventory [68] and the Actively Open Minded Thinking Scale [36,37], since these self-report measures afford useful insights into how participants experience their own cognitive processes. Without such measures we may conflate cognitive wastrels, whose performance could be ameliorated through training, with genuine cognitive misers, who elect not to be analytic and who may not be amenable to training interventions.
